# Effect of early pulmonary rehabilitation therapy on the pulmonary function of patients with stroke-associated pneumonia and analysis of its effectiveness

**DOI:** 10.12669/pjms.40.7.8113

**Published:** 2024-08

**Authors:** Zhanjun Liao, Feiwei Liao

**Affiliations:** 1 Zhanjun Liao, Rehabilitation Medicine Department, Liuzhou People’s Hospital, Liuzhou, Guangxi, China; 2 Feiwei Liao, Rehabilitation Medicine Department, Liuzhou People’s Hospital, Liuzhou, Guangxi, China

**Keywords:** Early pulmonary rehabilitation, Stroke-associated pneumonia, Pulmonary function, The National Institute of Health Stroke Scale

## Abstract

**Objective:**

This study aimed to evaluate the clinical effectiveness of early pulmonary rehabilitation (PR) treatment methods for stroke-associated pneumonia (SAP).

**Methods:**

This is a prospective, randomized controlled intervention study. Eighty SAP patients admitted to the rehabilitation department of Liuzhou People’s Hospital from June 2020 to December 2021 were selected and divided into an intervention group (40 cases) and a control group (40 cases) using the random number table approach. Patients in both groups received conventional treatments. Patients in the intervention group received early PR interventions, while patients in the control group received conventional rehabilitation treatment. The clinical effectiveness and pulmonary function indexes were compared.

**Results:**

The intervention group showed shorter durations of stay in the intensive care unit, relief from symptoms, and mechanical ventilation compared to the control group following treatment (P < 0.05). The total clinical efficacy after intervention in the treatment group was 82.50%, significantly higher than the control group’s rate of 47.50% (P < 0.05). After 21 days of treatment, the forced expiratory volume in one second (FEV1)/predicted value in the intervention group was 64.92 ± 8.53, while it was 52.69 ± 7.08 in the other group. The FEV1/forced vital capacity in the intervention group was 59.73 ± 7.18, compared to 50.36 ± 6.54 in the control group. The intervention group had a clinical pulmonary infection score of 2.62 ± 1.13, while the control group had a score of 4.17 ± 2.08. The intervention group showed a significant improvement in lung function, with statistically significant differences compared to the other group (P < 0.05). Moreover, the intervention group had significantly lower levels of interleukin-4 and interleukin-10 compared to the control group, with statistical significance (P < 0.05). The average National Institute of Health Stroke Scale score of the intervention group was significantly lower than that of the control group in the second and third weeks after treatment (P < 0.05).

**Conclusion:**

Early comprehensive pulmonary rehabilitation can significantly enhance the pulmonary function and respiratory status of SAP patients and facilitate the early recovery of brain function. This approach archives significantly better outcomes compared to conventional PR; therefore, it is worth clinical implementation.

## INTRODUCTION

In China, there has been a noticeable increase in the occurrence of acute stroke in recent times, and stroke-associated pneumonia (SAP) is a common complication of stroke as it has an incidence ranging from 2.4% to 47.0%.^1^,^2^ Studies have shown that SAP occurs in the early post-stroke period, especially within the first three days, and is closely related to factors such as swallowing dysfunction and immune deficiency. SAP is defined as a pulmonary infection that occurs in the acute and post-stroke phases of stroke patients.^3^ It often leads to an extended duration of hospital stay, increased medical costs, and serious threats to patients’ lives and health. It has been reported that the incidence of SAP increases annually as stroke patients age.^4^ Therefore, clinical interventions for SAP have attracted the attention of medical experts.

Pulmonary rehabilitation (PR) is an intervention for people with chronic respiratory diseases who experience symptoms of discomfort, reduced daily activities, or decreased ability to perform everyday tasks.^5^ Since the introduction of definition of PR in 1994, the application of PR has gradually become more popular and widely accepted in clinical practice. In 2013, the American Thoracic Society (ATS) and the European Respiratory Society (ERS) jointly published a statement on “Evidence-Based Guidelines for PR”, which updated the definition of PR.^6^ The American Heart Association (AHA)/American Stroke Association (ASA) recommended the necessary PR for stroke patients, including passive vibration for sputum removal, active exercise, and respiratory muscle exercises in the 2016 guidelines for post-stroke rehabilitation of adults.^7^

In recent years, scholars in China and abroad have conducted numerous studies related to PR. A study suggests that bedridden patients with SAP for an extended period may experience a decrease in lung and thoracic expansion capacity, as well as respiratory muscle weakness, resulting in reduced oxygenation index and breathing difficulties. These symptoms can affect the patient’s oxygen supply status, leading to pulmonary infections and increased mortality rates. PR training involving exercises targeting respiratory muscles, chest loosening techniques, and physical activities can enhance the respiratory ability of stroke patients with SAP, improve the oxygenation index, promote coughing and sputum clearance, alleviate symptoms of breathing difficulties in SAP patients, and facilitate overall functional recovery.^8^ Domestic guidelines suggest that early administration of PR after a stroke can mitigate the occurrence of aspiration and hypostatic pneumonia, minimize the likelihood of requiring tracheotomy, and decrease the duration of hospitalization.^9^ However, in actual clinical practice, the implementation of PR therapy by healthcare professionals is not sufficient. Some studies suggest that an imbalance in resource distribution and a single mode in the PR environment lead to deviations in patients’ internal expectations, reducing their psychological motivation to actively engage in PR.

Although the treatment of SAP with traditional PR measures in China has achieved some success, they are fragmented and relatively independent. There is a lack of systematic framework and standardized protocols, resulting in a less satisfactory rehabilitation effect. Therefore, traditional pulmonary rehabilitation treatment methods are no longer sufficient to meet the growing needs of patients. Based on this, 80 patients with SAP were admitted to the rehabilitation department of our hospital from June 2020 to December 2021. They were treated and followed up to explore the clinical effect of early comprehensive PR therapy in SAP. This work aims to provide a reference for future clinical work.

## METHODS

### Research Subjects

Eighty patients with SAP admitted to the rehabilitation department of our hospital between June 2020 and December 2021 were selected and divided into two groups, each consisting of 40 cases, using the random number table.

### Ethical Approval

This study was approved by the ethics committee of Liuzhou People’s Hospital (No.KY-2023-098 dated on February 15, 2023).

### Inclusion Criteria:

- Stroke diagnosis conformed to the diagnostic criteria of the Chinese Classification of Cerebrovascular Diseases (2015),^10^ and SAP met the diagnostic criteria described in the Chinese Expert Consensus on the Diagnosis and Management of SAP.^11^
- Without lung infection.
- Informed consent of the patient and the patient’s family.

### Exclusion criteria:

- Patients whose families requested to withdraw midway or who could not cooperate for various reasons.
- With severe cardiopulmonary, hepatic, renal, and other organ insufficiency.
- With malignant neoplastic diseases.
- With autoimmune diseases.
- With a history of mental illness, deafness, or muteness.
- Both groups of patients received conventional drug treatment. The control group underwent routine rehabilitation therapy, which included:
- health education;
- positioning and active/passive movement of the affected limbs;
- lower limb robot training;
- transfer training;
- sitting and standing balance training;
- daily life activity capacity training. Each session lasted for 30 minutes, once a day.

In the intervention group, early PR interventions were carried out. The first intervention was relaxation training.^12^ The initial training focused on thoracic relaxation. When the patient remained supine, the therapist placed one hand under the patient’s shoulder and pushed the shoulder joint upward with the wrist. Simultaneously, the therapist placed the other hand above the pelvis to assist the patient in moving the thorax upward. The second training was intercostal muscle relaxation training. When the patient remained supine, the therapist moved one hand along the rib and positioned the other hand near the rib. He/she squeezed and vibrated the rib when the patient exhaled and removed compression when the patient inhaled, twice a day. The second intervention session focused on respiratory muscle. The patient received abdominal respiratory rehabilitation intervention, pursed-lip respiratory rehabilitation intervention, external diaphragmatic pacemaker stimulation training, and three-ball respiratory training, 20 minutes each time, twice a day. The third intervention was motor rehabilitation training.^13^ The corresponding exercise rehabilitation intervention program was designed for each patient within 24 hours after admission based on disease assessment. The rehabilitation therapist taught the patient how to perform passive limb exercises as part of the intervention. If the patient was conscious and able to cooperate, and his Richmond Agitation-Sedation Scale (RASS) score was between -1 point and one point, the therapist assisted him in receiving stepwise passive limb exercise intervention to passively exercise upper and lower limb joints. As the patient’s condition improved, the patient was gradually permitted to engage in active weight-bearing exercises for both upper and lower limb joints independently. Initially, the patient performed semi-sitting exercises in the bed, followed by sitting exercises in a bedside chair, sitting weight-bearing exercises, assisted standing exercises, independent standing exercises, and walking exercises, each lasting 20 minutes, twice daily.

### Observation indicators:

**-** Pulmonary function: Before treatment and three weeks after treatment, forced expiratory volume in the first second (FEV1), FEV1/predicted value (FEV1/Pre), forced vital capacity (FVC), and the ratio of FEV1 to FVC (FEV1/FVC) were measured using a pulmonary function device (Master Screen PFT) in both groups. Measurements were repeated more than three times, and the best value was selected. The higher the score, the more severe the condition.
- Clinical pulmonary infection score (CPIS): The CPIS was used for a comprehensive assessment. - Rehabilitation indicators: the duration of intensive care unit (ICU) stays, mechanical ventilation, and symptom relief for both groups were recorded.
- Inflammatory factor assay: a fasting venous blood of 3ml was collected from every patient in both groups before treatment and the following morning after three weeks of treatment, and the concentrations of interleukin-4 (IL-4) and interleukin-10 (IL-10) were quantified using enzyme-linked immunosorbent assay.-
- The National Institute of Health Stroke Scale (NIHSS) score: The NIHSS score was employed to measure the recovery of brain function in SAP patients. The baseline level reflected the severity of the stroke. Following treatment, the impact of the intervention was assessed based on consciousness, gaze, facial palsy, visual field, limb ataxia, limb movement, sensory perception, language abilities, dysarthria, neglect, etc. The total score was 42 points; the higher the score, the more severe the neurological damage.^14^
- Clinical efficacy assessment:^15^ Cure was defined as complete disappearance of clinical symptoms, normalization of blood routine, and complete absorption of inflammation shown on lung computed tomography (CT); significant improvement was defined as a significant reduction in clinical symptoms, normalization of blood routine, and partial absorption of inflammation shown on lung CT; ineffective was defined as no obvious improvement or even worsening trend in clinical symptoms, blood routine, and lung CT. The total effective rate = (number of cured cases + number of significantly improved cases)/total number × 100%.

### Statistical analysis

The statistical package SPSS version 24.0 was used for data analysis and processing. All measurement data were presented as (*χ̅*±*S*)and the independent sample t-test was used for comparison between groups. Count data were expressed as n(%), and the chi-square test was used for comparison between groups. The difference was considered statistically significant when P < 0.05.

## RESULTS

A comparison of general data between the two groups demonstrated no statistically significant differences (P > 0.05), indicating comparability (Table-I). The durations of intensive care unit (ICU) stay, mechanical ventilation, and symptom relief in the intervention group were all shorter than those in the control group, and the differences were statistically significant (P < 0.05) (Table-II).

**Table-I:** Comparison of general data between two groups.

Group		Intervention group (n=40)	Control group (n=40)	t/X^2^	P
Age		58.95±9.48	58.69±9.36	1.083	> 0.05
Gender	Male	27	30	0.614	> 0.05
	Female	13	10		
Diagnosis	Cerebral hemorrhage	25	31	0.735	> 0.05
	Cerebral infarction	15	9		

**Table-II:** Clinical indicators of rehabilitation between the two groups.

Group	Intervention group (n=40)	Control group (n=40)	t	P
Duration of ICU stay	14.63±2.78	19.61±3.24	6.671	< 0.05
Duration of mechanical ventilation	7.54±1.65	10.12±2.19	5.319	< 0.05
Duration of symptom relief	4.54±0.81	7.57±1.18	11.274	< 0.05

After three weeks of treatment, the total efficacy of clinical treatment in the intervention group was significantly higher than that in the control group (P < 0.05) (Table-III).

**Table-III:** Comparison of clinical efficacy between two groups [n(%)].

Group	Intervention group (n=40)	Control group (n=40)	t	P
Cured	4 (10.00)	1 (2.50)	11.917	< 0.05
Significant improvement	29 (72.50)	18 (45.00)		
Ineffective	7 (17.50)	21 (52.50)		
Total effective rate	33 (82.50)	19 (47.50)		

Before treatment, there was no statistically remarkable difference in pulmonary function indicators between the two groups (P > 0.05). The pulmonary function after three weeks of treatment was significantly different from that before treatment in both groups (P < 0.05). The improvement of pulmonary function in the intervention group after treatment was better than that in the controls, and the difference was statistically significant (P < 0.05) (Table-IV).

**Table-IV:** Pulmonary function and CPIS before and after treatment in the two groups.

Group		Intervention group (n=40)	Control group (n=40)	t	P
FEV1/Pre/%	Before treatment	42.37±9.18	45.84±8.57	0.073	> 0.05
	After three weeks of treatment	64.92±8.53	52.69±7.08	3.715	< 0.05
FEV1/FVC/%	Before treatment	41.58±5.07	40.22±6.91	0.068	> 0.05
	After three weeks of treatment	59.73±7.18	50.36±6.54	3.154	< 0.05
CPIS	Before treatment	7.27±2.88	7.46±2.13	0.623	> 0.05
	After three weeks of treatment	2.62±1.13	4.17±2.08	3.891	< 0.05

Before treatment, the differences in IL-4 and IL-10 between the two groups were not statistically remarkable (P > 0.05). The differences in IL-4 and IL-10 in the same group before treatment and after three weeks of treatment were statistically significant (P < 0.05). The reduction of inflammation in the intervention group was more significant than that in the control group after treatment, and the difference was statistically remarkable (P < 0.05) (Table-V).

**Table-V:** Inflammatory factors before and after treatment in the two groups.

Group		Intervention group (n=40)	Control group (n=40)	t	P
IL-4 (ug/mL)	Before treatment	141.76±26.08	135.92±28.37	0.063	> 0.05
	After three weeks of treatment	69.75±18.25	92.32±19.51	5.234	< 0.05
IL-10 (ug/mL)	Before treatment	210.18±26.36	220.57±24.12	0.075	> 0.05
	After three weeks of treatment	150.39±24.18	189.24±36.66	2.891	< 0.05

**Fig.1:**
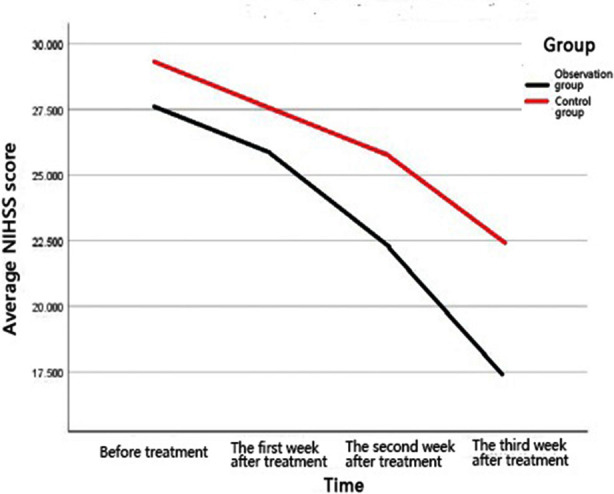
NIHSS score trends before and after treatment between the two groups of patients.

The difference in the NIHSS score between the two groups before treatment and at the first week after treatment was not statistically significant (P > 0.05); the difference in the recovery of brain function between the first week and third week after treatment was statistically significant (P < 0.05), and the NIHSS scores in the intervention group were significantly lower than those in the control group (Fig.1).

## DISCUSSION

The results of this study demonstrated that the intervention group exhibited not only overall improvements in lung function and levels of inflammatory factors compared to the control group, but also a higher total effective rate of clinical treatment (P < 0.05). This suggested that early PR intervention, which introduced respiratory muscle strength training to enhance diaphragm function and lung tissue elasticity, relaxation training to increase chest mobility and compliance, as well as exercise rehabilitation training to promote improvement in patients’ physical condition, alleviated symptoms of SAP infection, improved lung function, and facilitated recovery in SAP patients. As the emphasis on quality of life in recent years increases, more clinical attention has been paid to the treatment of SAP, including airway management, nutritional support, cross-infection blockade, anti-infection therapy, etc.^16^,^17^ However, patients are passive recipients of these treatment measures, and treatment relies heavily on external drugs and assistive devices, which is not satisfactory for patients who have been bedridden for a long time after a stroke. Moreover, many SAP patients have significantly weakened lung function, and the recovery of patients with this disease cannot be effectively promoted by purely passive treatment.^18^,^19^ In this study, the treatment plan of the intervention group was designed based on the results of pulmonary function and comprehensive assessment, starting from simple pursed-lip breathing to abdominal breathing and from passive exercise, active muscle training, to endurance training. The whole treatment process followed the concept of gradual and individualized rehabilitation, which finally obtained good clinical efficacy, allowed the patients to get out of the ICU ward as early as possible, and reduced their mechanical ventilation duration, and this is in line with previous research findings.^20^,^21^

The respiratory system is the oxygen supply system of the human body. Active PR after stroke can improve pulmonary ventilation and air exchange, which can improve the ischemic and hypoxic state of the brain, and has a significant improvement and promotion effect on both cardiopulmonary and brain function of patients; however, there are few clinical reports on the specific effects of PR on brain function in SAP patients. This paper introduced the NIHSS score to evaluate the recovery of brain function in stroke patients after a period of PR, which supplements the missing content in this research area.^22^,^23^ It was found from the research results that there was no remarkable difference in the recovery of brain function between the two groups on the 7th day after early comprehensive PR treatment in SAP patients, and the recovery of brain function was much better in the intervention group than in the control group at the 14th and 21st day after treatment, which showed that the difference in recovery of brain function was not highlighted in the early stage of treatment but gradually became prominent in the later stage of treatment, which is consistent with the pathophysiological situation--slow recovery after neurological damage.^24^

### Limitations

It includes a small number of subjects. In future, there is a need to conduct large-scale clinical trials to further explore PR exercise training to actively identify effective pulmonary rehabilitation exercise training that is easier to adhere to to facilitate clinical promotion and implementation.

## CONCLUSION

In conclusion, early pulmonary rehabilitation intervention in the treatment of SAP has satisfactory clinical effects as it can effectively relieve patients’ infection status, improve pulmonary function, significantly reduce ICU hospitalization time and mechanical ventilation time, and promote the early recovery of brain function. Its effect is significantly better than traditional pulmonary rehabilitation treatment, making it a more effective, reasonable, and meaningful approach worth promoting.

### Authors’ Contribution:

**ZJL:** Study design, data collection and analysis.

**ZJL** & **FWL:** Manuscript preparation, drafting and revising.

**ZJL:** Review and final approval of manuscript.

**ZJL:** Is responsible and accountable for the accuracy and integrity of the work.
